# Burden and cost of multiple sclerosis in Brazil

**DOI:** 10.1371/journal.pone.0208837

**Published:** 2019-01-23

**Authors:** Gisela Kobelt, Vanessa Teich, Marcela Cavalcanti, Ana Maria Canzonieri

**Affiliations:** 1 European Health Economics, Stockholm, Sweden; 2 Sense Company, São Paulo, Brazil; 3 Associação Brasileira de Esclerose Múltipla (ABEM), São Paulo, Brazil; Universita degli Studi di Napoli Federico II, ITALY

## Abstract

**Background:**

The objective of this study was to estimate costs to society and patients’ quality of life (QoL) at all levels of disease severity (measured with the Expanded Disability Status Scale, EDSS) in Brazil.

**Methods:**

The study was part of an international, cross-sectional burden-of-illness study carried out in collaboration with national MS patient organizations. All information was collected directly from patients using a validated questionnaire. Direct costs were estimated both from societal and payer perspectives, while total costs are presented as societal costs.

**Results:**

The survey included 694 patients (response rate 21%; mean age 40.8 years). 95% of patients were of working age, and around half were working. The mean EDSS score was 3.2 (62.5% of patients with EDSS <3). Relapses were reported by 18.9% of patients. Fatigue affected almost all patients (94%) regardless of EDSS level, and cognitive difficulties were reported by 69.1% of patients. Mean utility ranged from 0.77 at EDSS 0 to negative values at EDSS 9, with a mean score of 0.58; utility was affected by relapses. Total mean annual cost was R$33,872 (€ 8,000) per patient in the societal perspective, with direct costs representing 81% (R$ 27,355, € 6,500). Direct costs for the payer amounted to R$ 16,793 (€ 4,000)/patient.

**Conclusions:**

This study included a population with relatively mild and early disease, with a majority of patients with relapsing disease and thus on DMD treatment. It is not possible to conclude directly on the total cost of MS in Brazil. Nevertheless, resource quantities used, QoL and MS symptoms are very similar to what was seen in the European survey.

## Introduction

Multiple sclerosis (MS) is a chronic, autoimmune inflammatory disease that affects the central nervous system (CNS) [1]. It is a complex and heterogeneous condition that affects young adults, with an average of two women for each man [2]. Brazilian epidemiological data show a prevalence of 8.69 per 100,000 population, with however a large regional variation (from 1.36 to 27.2 per 100,000 inhabitants in the Northeast and South regions, respectively) [1,3].

At onset MS presents with clinically isolated neurological events (CIS) that develop into relapsing-remitting MS (RRMS) in the majority of patients. Within 10–15 years after diagnosis, the majority of patients convert to secondary progressive MS (SPMS). Primary progressive MS at onset is present in 5–10% of patients. Currently, pharmacological treatment with disease modifying drugs (DMDs) is initiated at the first CIS in many countries to delay diagnosis, and after confirmed diagnosis everywhere. The aim of treatment is to reduce the number and intensity of exacerbations and thereby delay progression to disability [4].

The Brazilian public healthcare system offers treatment with glatiramer acetate, betainteferon 1a and 1b, fingolimod and natalizumab for the treatment of RRMS [5]. Azatioprine is recommended in case of poor adherence to parenteral forms and methylprednisolone is reimbursed for the treatment of relapses. In 2017, two additional drugs (teriflunomide and dimethyl fumarate) have received a positive recommendation from the National Commission for Incorporation of Health Technologies (CONITEC) [6,7], the Health Technology Assessment Agency in charge of evaluating new technologies for incorporation into the Brazilian public health care system (SUS). The updated guideline has been published in April 2018 [8], but the two drugs are currently not yet available to patients. Although DMDs are reimbursed since 2010, their impact on costs and outcomes has not been evaluated, mainly due to the lack of real life follow-up data. Also, only a few studies have attempted to measure costs due to MS or the impact on patients’ quality of life in Brazil [9,10].

This Brazilian study was part of an international, prevalence-based, cross-sectional, observational burden-of-illness study, carried out in collaboration with national MS patient organizations. The European study included 16 countries and enrolled 16,800 patients, and overall and country specific results have been published elsewhere [11]. The primary objective was to estimate all resource consumption and production losses caused by MS at different stages of the disease. Additional objectives were to estimate the impact of the disease on quality of life (utility), and investigate the level of fatigue and cognitive difficulties experienced by people with MS.

## Materials and methods

This study followed the same methodology as the European survey [11,12], and methods are therefore only summarized here.

All information was collected directly from patients using a questionnaire, validated by a clinical expert and a patient association (Associação Brasileira de Esclerose Múltipla, ABEM). Ethical approval for the study was obtained from the Universidade de Santo Amaro, São Paulo, Brazil. The patient association invited a total of 3,226 patients to participate and complete the questionnaire anonymously either on paper or on-line. The identity of the respondents was thus not known, making it impossible to verify the answers or complete missing data.

Resources used or lost were assessed with questions related to all health care consumption (hospitalizations, consultations, tests, prescription and over-the-counter medications), services (home care, transportation), investments (devices and changes to the home or car), informal care provided by family and production losses (sick absences and early retirement due to MS). Time periods for the questions were varied in order to ensure the best possible recall [11]. The average cost of a relapse was estimated as the difference 3-month costs between patients with and those without a relapse, based on the assumption that a relapse generally didn’t exceed 3 months. DMDs were excluded from the calculation as it is unlikely that these long-term treatments change during a short-term relapse. In addition, the calculation was limited to patients with an EDSS score of 0–5, where RRMS is most frequent and relapses more noticeable.

Disease information collected included the number of relapses and the level of disability using patient-assessed EDSS (Expanded Disability Status Scale [13]). MS related symptoms such as fatigue and cognitive impairment were assessed with visual analogue scales (VAS, 1 no problem, 10 severe problems) preceded by a binary question. Health related quality of life was assessed with the EQ-5D-3L converted into utilities using the Brazilian tariff [14].

In view of the cross-sectional design, only descriptive analyses are presented. Overall costs were estimated in the societal perspective, i.e. regardless of who ultimately bears the cost. However, unit costs for health care resources and services were taken from the national databases of the health insurer (SUS), and direct costs represent thus payer costs [15–17]. Patients’ out-of pocket costs were subsequently added. Informal care was considered a direct cost, as in the absence of family help the health care system would have to provide the service. The cost of informal care was calculated from the average national disposable income. Production losses (indirect costs) were estimated using the Brazilian GDP per capita to estimate the economic impact of workdays lost [18,19].

The study aimed at estimating the burden of the disease by level of disability rather than for a representative population. Therefore, an entirely anonymous recruitment process was preferred to a controlled enrolment, and our results therefore do not represent a prevalence sample. Costs can thus not be extrapolated to national costs without weighting by the actual disease severity distribution in Brazil.

## Results

A total of 694 patients provided valid answers during the period between April 2016 and December 2017, a response rate of 21.3%. Participants came from all Brazilian regions, with however an overrepresentation of the Southeast region (70.2%).

### Demographics

Table 1 presents the characteristics of the sample. The mean age at completion was 40.8 years (range 19–78) and the majority lived with family or friends (91.6%). Women represented 78.6%. A large proportion reported a university degree (62.1%) while only 2.7% stated that they had only primary school education.

**Table 1:** Sample demographics and disease information.

Characteristics	n (%)
Sample (N)	**694 (100%)**
Mean age (SD)	40.8 (11.3)
Proportion women	78.7%
Proportion living with family/friends	91.6%
Geographical area	
North	1.6%
Northeast	6.6%
Middle West	7.5%
Southeast	70.2%
South	14.1%
Education	
Primary school	2.7%
High school degree or professional diploma	33.7%
University degree	62.1%
Missing	1.4%
Employment	
Patients of working age ^a^	657 (94.7%^b^)
Total currently employed or self-employed	321 (46.3%^b^)
Working age, employed or self-employed	321 (48.9%^c^)
Working full time	163 (50.8%^d^)
On long-term leave (>3 ⩽ 12 months)	37 (11.5%^c^)
Sick leave (past 3 months)	73 (22.7%^c^)
Not working due to MS	254 (38.7%^b^)
Permanent sick leave/invalidity pension	145 (22.1%^b^)
Disease information	
Mean age at diagnosis (SD)	32.4 (9.6)
Mean age at first symptoms (SD)	27.8 (9.3)
Mean EDSS (SD)	3.2 (2.5)
Mild MS (EDSS 0–3)	434 (62.5%)
Moderate MS (EDSS 4–6.5)	177 (25.5%)
Severe MS (EDSS 7–9)	83 (12.0%)
Proportion with RRMS	519 (74.8%)
Proportion with relapses in the last 3 months	131 (18.9%)
Proportion using DMTs	403 (58.1%)

^a^by legal retirement age of 60/65 years for women and men, respectively;

^b^ of total sample (N = 694);

^c^ of patients of working age (N = 657);

^d^ of patients working (N = 321).

MS: Multiple Sclerosis; EDSS: Expanded Disability Status Scale; RRMS: relapsing-remitting multiple sclerosis; DMD: Disease modifying treatments

The mean EDSS score was 3.2 (SD 2.5), indicating a sample with predominantly mild disease: 62.5% had an EDSS score below 3 and 25.5% a score between 4 and 6.5, indicating that the study did not easily reach severe patients. Our results for the severe group at EDSS 7–9 have therefore to be considered with caution. This distribution of disease severity also explains the high proportion of patients with RRMS (74.8%). Relapses were reported by 18.9% of patients, but 14.7% were unsure, and we assumed that these patients had no relapse.

### Employment

**Fig 1:**
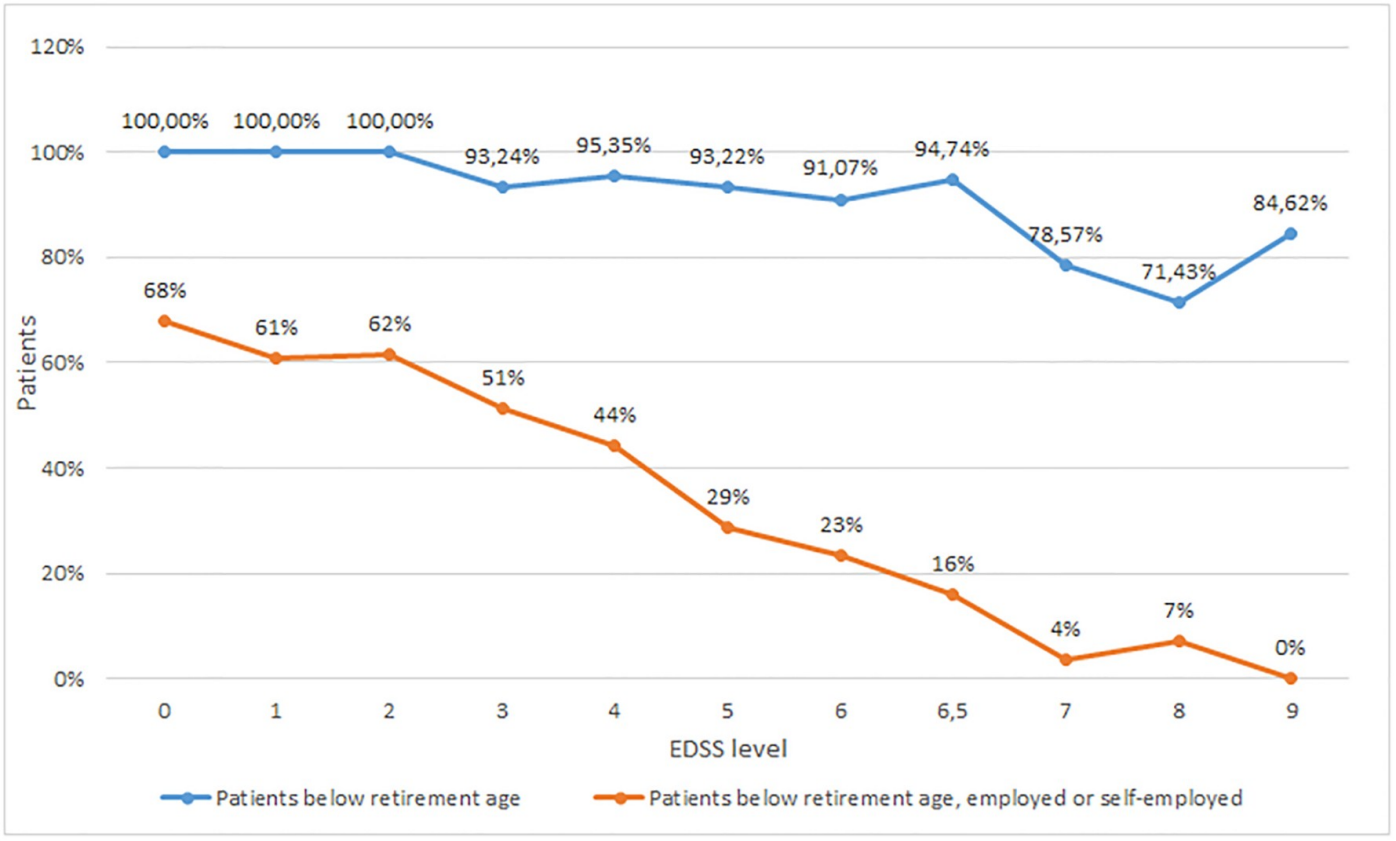
Proportions of patients below retirement age and employed/self-employed. The vast majority of patients in the sample were of working age (95%). Workforce participation decreased rapidly with increasing disability (calculated as the proportions of patients of working age).

Ninety-five percent of respondents were below the official retirement age (60 years for women and 65 years for men). However, half of these patients (51.1%) were not working, most of them due to MS (68.7%). Permanent sick leave/invalidity was the most common situation. Among employed or self-employed patients, 50.8% worked full-time while 19% had reduced their working hours due to MS. Sick-leave during the past 3 months was reported by 22.7% (mean 47.7 days), and 11.5% had been on long-term sick-leave (mean 17.9 months). Work force participation for patients of working age decreased from 68% at EDSS 0 to 0% at EDSS 9, with a marked drop at EDSS 4 (Fig 1).

The effect of MS on work productivity while at work was measured using a VAS (ranging from 0 = health problems had no effect on my work to 10 = health problems completely prevented me from working). The mean VAS score was 3.7 (SD 2.9), with the reasons for a negative effect being fatigue (59.8%), low mood (37.3%), difficulty thinking (29.9%) and physical pain (28.0%).

### Quality of life, utilities, fatigue and cognition

**Fig 2:**
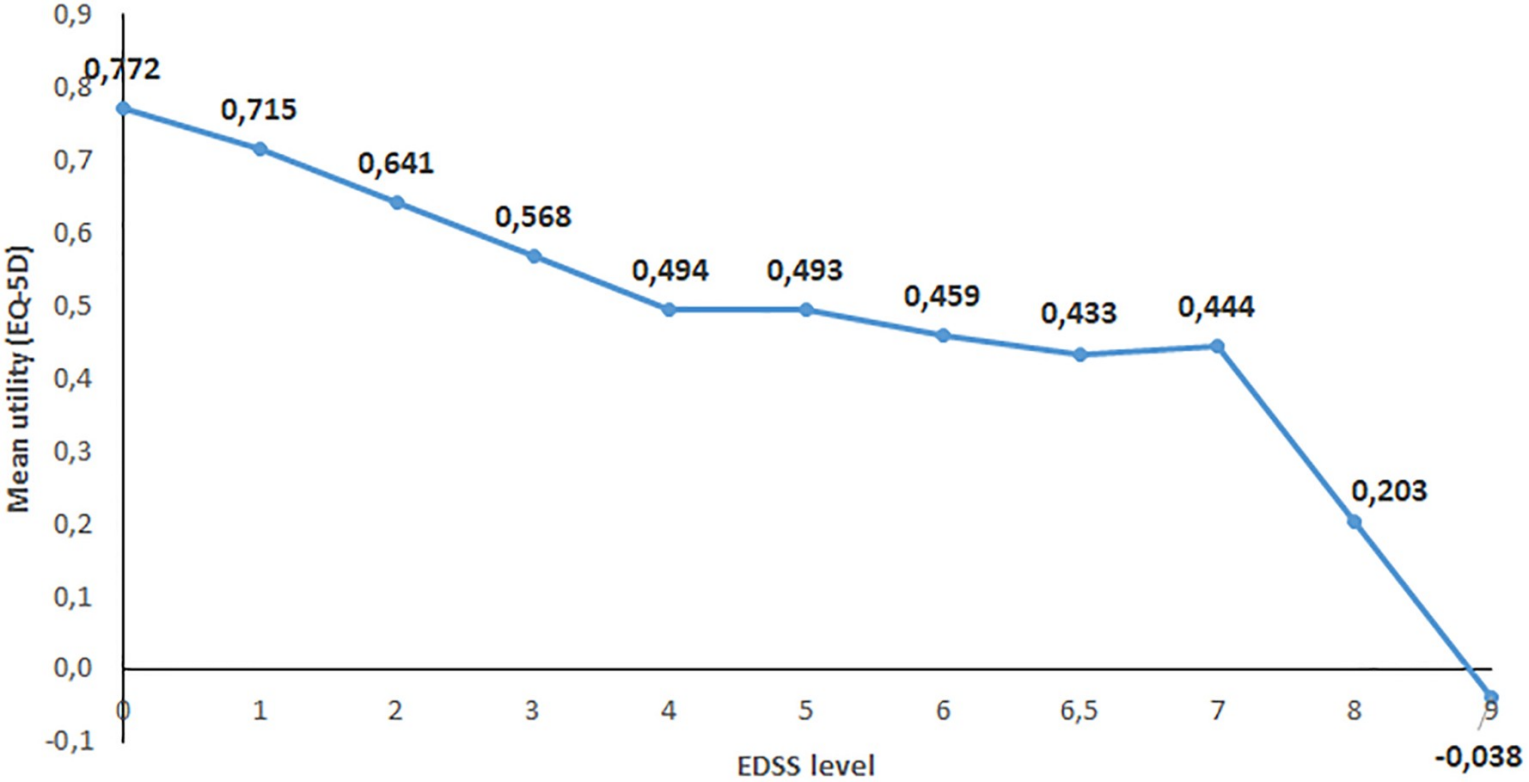
Effect of disability on utility. Utility is calculated with the EQ-5D and expressed as patients’ preferences of given health states on a scale between 1 = full health and 0 = death (with negative values possible representing health states that are judged worse than death). The calculations use Brazilian population values for health states. Disability is expressed as EDSS scores.

Mean utility using the Brazilian EQ-5D value set was 0.576 for the entire sample. Males had slightly lower mean utility than females (0.540 versus 0.586). Utility decreased with increasing disability (Fig 2) and was affected by relapses. Patients who had experienced a relapse had a utility score of 0.585 compared to 0.675 for those without a relapse (EDSS 0–5).

Fatigue affected almost all patients (94%, 2% missing) at a relatively high and almost constant level (mean VAS score 5.7, SD 2.7) regardless of disability. Cognitive difficulties were reported by 69.1% of the sample, with a mean VAS score of 4.95 (SD 2.1). Impairment increased slightly with increasing EDSS level from 4.6 in mild disease to 5.8 in severe disease.

### Costs

**Fig 3:**
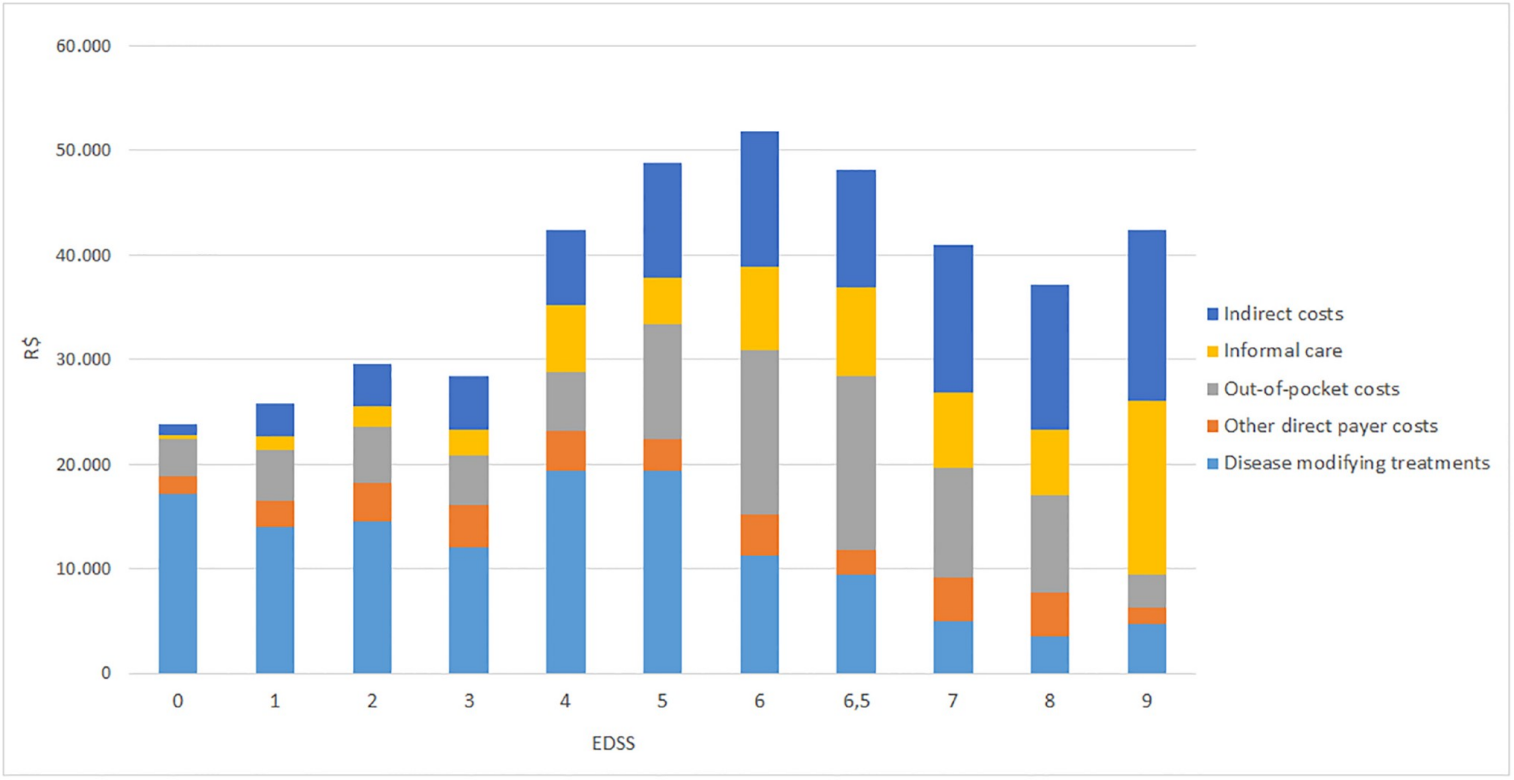
Annualized mean total costs by EDSS level (Brazilian Reais 2016. R$). Total costs are presented as societal costs by disability (Expanded Disability Status Scale. EDSS). For total direct costs, a distinction was made between costs to payers (the health care system) and costs to patients and families (out-of pocket costs. informal care). The highest costs are reached at EDSS 6, where after DMTs costs decrease markedly as patients convert to progressive disease. However. The group of patients at 7–9 is small and results have to be considered with caution.

Table 2 presents the number of patients using selected important resources and the intensity of usage, as well as related costs. Table 3 and Fig 3 present total societal costs.

**Table 2:** Use and cost of selected resources (Brazilian Reais. 2016).

Selected resources	Proportion of the sample using the resource	Mean annual cost (SD)	Median cost [95% CI]
		R$ 2016	R$ 2016
**Hospitalizations**	**146 (21%)**	**838 (3.148)**	**0 [0–9.209]**
Inpatient care (mean 6.5 days)	78 (11%)	747 (3.016)	0 [0–8.730]
Day-case admissions (mean 3.5 times)	95 (14%)	92 (401.24)	0 [0–798]
**Consultations (past 3 months)**	**562 (81%)**	**197 (495)**	**80 [0–1.304]**
Neurologist	489 (70%), mean 2.8 times	79 (237)	40 [0–387]
General practitioner	41 (6%), mean 2.5 times	6 (61)	0 [0–40]
MS nurse	29 (4%), mean 1.7 times	2(11)	0 [0–25]
Physiotherapist	159 (23%), mean 17 sessions	46 (184)	0 [0–502]
Psychologist/Counselor	96 (14%), mean 9.3 times	21 (128)	0 [0–400]
**Tests and investigations (past 3 months)**	**434 (63%)**	**870 (1.025)**	**49 [0–2.664]**
MRI (brain)	301 (43%)	466 (533)	0 [0–1.075]
MRI (spine)	218 (31%)	337(499)	0 [0–1.07]
Blood test	363 (52%)	22 (55)	16 [– 132]
**Medications (past month)**	**403 (58%)**	**14.849 (21.155)**	**19.820 [0–34.731]**
Disease-modifying treatments	403 (58%)	13.634 (20.340)	19.820 [0–28.423]
*EDSS 0–3*	280 (69.5%)	14.571 (17.037)	19.820 [0–28.423]
*EDSS 4–6*.*5*	104 (25.8%)	15.758 (28.811)	19.820 [0–28.423]
*EDSS 7–9*	19 (4.7%)	4.202 (8.817)	0 [0–28.423]
Corticosteroids	97 (14%)	1.145 (5.774)	0 [0–6.888]
Walking, spasticity, pain treatments	153 (22%)	38 (131)	0 [0–570]
Urological treatments	24 (3%)	2 (31)	0 [0–0]
Fatigue treatments	61 (9%)	5 (21)	0 [0–38]
Depression treatments	135 (19%)	25 (61)	0 [0–235]
**Investments. equipment and aids (past 12 months)**	**142 (20%)**	**25 (279)**	**0 [0–80]**
**Assistance (home care. transportation) (past month)**	**65 (9%)**	**12 (150)**	**0 [0–0]**
**Out-of-pocket expenses**	**474 (68%)**	**7.018 (18.213)**	**913 [0–82.034]**
Consultations	139 (20%)	2.323 (9.487)	0 [0–23.220]
Non-prescription medicines	402 (58%)	2.725 (11.079)	390 [0–17.446]
Assistance	28 (4%)	378 (2.570)	0 [0–5.448]
Investments	133 (19%)	1.593 (9.005)	0 [0–20.000]
**Informal care (past month)**	**210 (30%)**	**3.544 (8.306)**	**0 [0–30.407]**
EDSS 0–3 (13 days. 6.6 hours/day)	81 (12%)	1.466 (872)	833 [83–2.499]
EDSS 4–6.5 (17 days. 7.4 hours/day)	94 (14%)	6.475 (989)	1.250 [167–2.499]
EDSS 7–9 (23 days. 13.7 hours/day)	35 (5%)	8.154 (912)	2.499 [225–2.499]
**Production losses**		**6.518 [12.204]**	**0 [0–30.407]**
Short-term absence	73 (11%)	418 (1.551)	0 [0–7.602]
Long-term sick-leave 0–12 months	32 (5%)	841 (4.253)	0 [0–22.805]
Permanent sick-leave/Invalidity pension	120 (17%)	5.258 (11.507)	0 [0–30.407]

SD: Standard deviation; CI: Confidence interval; MS: Multiple sclerosis; MRI: Magnetic resonance imaging; EDSS: Expanded Disability Status Scale

**Table 3:** Total mean annual cost per patient (Brazilian Reais 2016).

	Annual costs per patient (R$ 2016)		
	Mean (SD)	Median [95% CI]	% of total societal costs
**Total costs, societal perspective**	**33.872 (34.807)**	**25.638 [0–126.789]**	
**Direct costs, societal perspective**	**27.355 (30.786)**	**22.786 [0–114.888]**	**80.8%**
Direct costs, payer perspective*	**16.793 (21.713)**	**19.915 [0–42.686]**	**49.6%**
Inpatient care	839 (3.148)	0 [0–9.209]	2.5%
Healthcare consultations	197 (495)	80 [0–1.304]	0.6%
Test and investigations	870 (1.025)	49 [0–2.664]	2.6%
Prescription medicines	1.216 (5.788)	0 [0–6.976]	3.6%
Disease-modifying treatments	13.634 (20.340)	19.820 [0–28.423]	40.3%
Investments, equipment and aids	25 (279)	0 [0–80]	0.08%
Assistance	12 (150)	0 [0–0]	0.04%
Patients’ out-of-pocket costs	7.018 (18.213)	913 [0–82.034]	20.7%
Informal care	3.544 (8.306)	0 [0–30.407]	10.5%
**Indirect costs**	**6.517 (12.204)**	**0 [0–30.407]**	**19.2%**
Short-term absence	418 (1.551)	0 [0–7.602]	1.2%
Long-term sick-leave 0–12 months	841 (4.253)	0 [0–22.805]	2.5%
Permanent sick-leave/invalidity pension	5.258 (11.507)	0 [0–30.407]	15.5%

*excluding patients’ out-of-pocket costs and informal care

The total mean annual cost in this sample was R$33,872 per patient in the societal perspective, of which direct costs represented 81% (R$ 27,355). Total mean annual direct costs for the payer (i.e. excluding out-of-pocket costs and informal care) amounted to R$ 16,793 per patient.

Direct costs were dominated by DMD usage and informal care. DMDs were used by 58% of the sample (70% in patients with mild disease at EDSS 0–3), at a mean annual cost per patient of R$ 13,634, or 81% of direct costs to the payer and 40.3% of total societal costs. Informal care was used by 30% of patients and amounted to a mean annual cost per patient of R$ 3,544, or 10% of costs to society. Dependence on informal care increased with increasing EDSS, with annual costs per patient increasing from R$ 1,466 in mild disease to R$ 8,154 in severe disease.

Patients’ mean out-of-pocket costs were estimated at R$ 7,018. A large proportion of these expenses were for over-the-counter medicines (39%) that were used by 58% of patients in the sample, while 33% represented co-payments for consultations and 23% investments in aids and adaptations.

Mean annual production losses amounted to R$ 6,517 per patient, or 19% of total societal costs. Costs were essentially due to long-term or permanent leave; short term sick leave represented only 6% of indirect costs.

**Fig 4:**
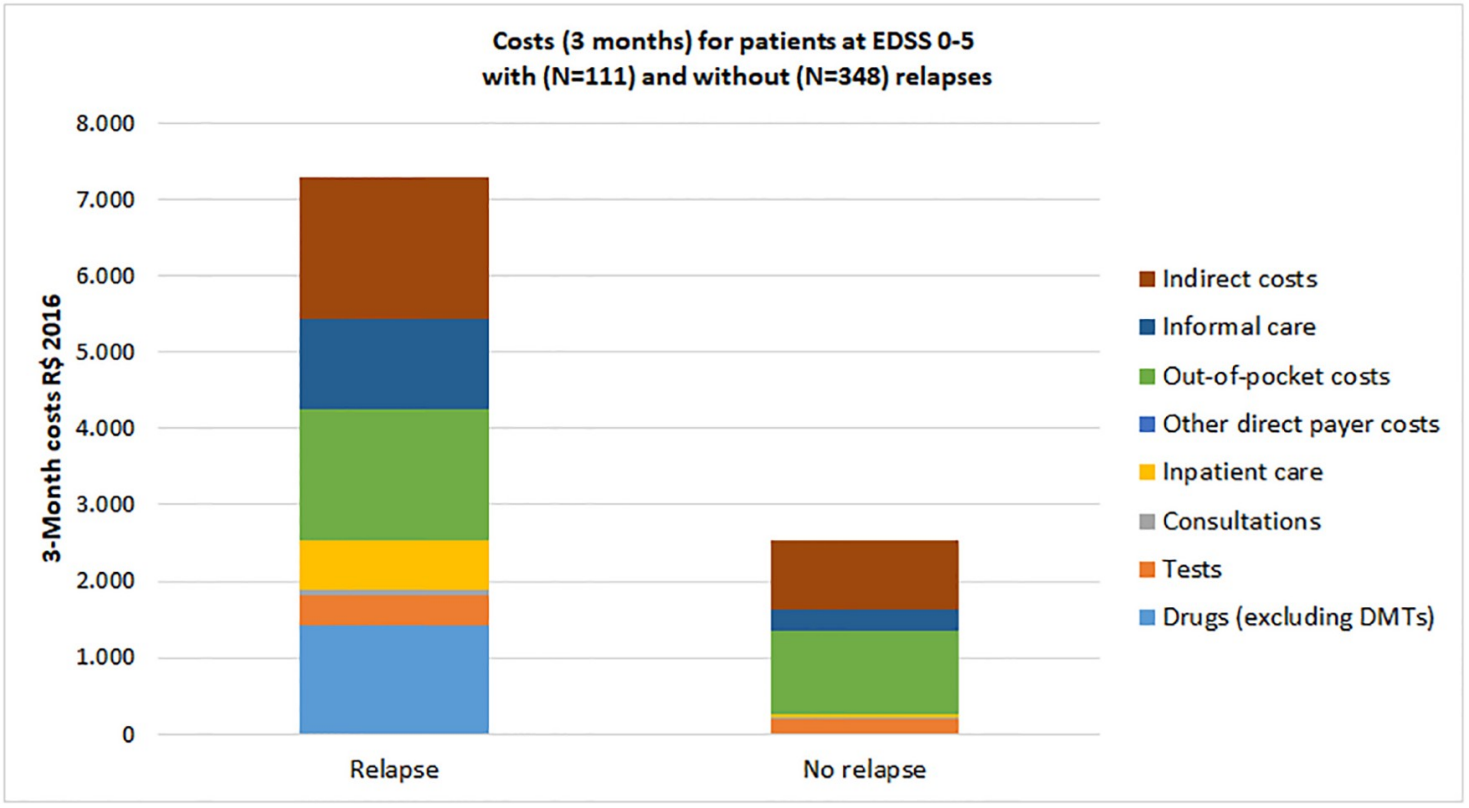
Costs for patients with and without a relapse over 3 months (Brazilian Reais 2016). The average cost of a relapse are calculated by comparison of costs of patients with and without relapses, limited to patients below EDSS 6 where relapses are more recognizable for patients. The cost of a relapse was estimated at R$ 4737.

The average cost of a relapse was estimated at R$ 4,737, based on mean 3-month costs excluding DMDs of R$ 7,283 and R$ 2,546 for patients with and without relapses, respectively. (Fig 4) All types of costs increased during a relapse, but most noticeably sick leave, informal care, prescription drugs, hospitalization and out-of-pocket costs. DMD usage was higher in the non-relapsing group, but our cross-sectional design does not allow concluding on causality.

## Discussion

This is the largest cost-of-illness study conducted so far in Brazil, including close to 700 patients. The response rate to the survey invitation of 21.3% is not unusual for this type of study with members of patient associations. Indeed, in the European study rates ranged from 13–29% (with two outliers at 38% and 70%). The study was designed to enroll sufficient patients at each EDSS level to allow analysis by disability levels, rather than a sample representative of the Brazilian MS population. Our results can therefore not be extrapolated to costs for MS in Brazil. Indeed, respondents were relatively young (mean age 40), had predominantly mild or early disease, with more than half of the patients at EDSS 0–2. As a consequence, the majority of patients had RRMS and DMD treatment was thus relatively high.

Also, the high percentage with a university degree (62%) and the dominance of participants from the Southeast Region, (Sao Paolo) indicates that the survey reached predominantly a young, well-educated urban population. According to the OECD, the national level of university education ranges from 9%-13% in the groups 55–64 year and 25–34 years, respectively (http://www.oecd.org/education/Brazil_EAG2013%20Country%20Note.pdfXX%). It is well possible that respondents did not distinguish between secondary and tertiary education, with the former estimated at 43% by the OECD. The dominance of the Southeast region (that represents 42% of inhabitants) may be explained by the fact that the patient association (ABEM) is located in Sao Paulo, but also the fact that the survey was carried out both on-line and with paper questionnaires.

With almost all respondents of working age (95%) and a mean EDSS in the sample of 3.2, it is not surprising that workforce participation was still at 49%, and half of employed patients worked full time. A study published in 2010 by Fragoso et al. [9] estimated workforce participation at 41%. The authors had performed telephone interviews with 96 randomly selected members of ABEM to evaluate the impact of the disease on patients’ professional life. Patients in this study were older than our sample (mean age 55), explaining the lower proportion of patients still working. The study found a significant negative correlation between disease duration and working regularly. In the European survey, the proportion of patients working in countries with a similar mean age (54–55 years) ranged from 31% in the Netherlands to 55% in Sweden. In countries with patients of a similar age as our Brazilian sample (38–42 years), the proportions ranged from 45% in Spain to 59% in Poland [11].

DMDs are indicated for patients with RRMS and the high proportion of patients with RRMS in our study explains the high usage (58% of patients). DMD treatment decreased with increasing EDSS. Overall, usage was similar to what was found in Eastern European countries (54%-58%), but considerably lower than in the Southern European countries (69%-79%). These comparisons are however indicative only: DMD use depends on a number of factors such as the age of the sample, the mean EDSS score, access (administrative hurdles), price and medical traditions.

Most patients with MS in Brazil use the public healthcare system, where medication, hospitalization and other services as consultations or personal assistance are available. We therefore present costs for these health care services separately, in the perspective of the payer, excluding patients’ out-of-pocket costs and informal care. Total costs are presented in the societal perspective, including all costs. This has the advantage to inform on the overall impact of people with MS, but also consider more specifically insurance costs. In these calculations, it is important to consider the number of patients who use a given resource. Consumption is generally extremely skewed, with few patients using certain resources intensively, with a very high cost. This can easily be observed in Tables 2 and 3 where the median costs for most resources are zero. We have therefore presented both the proportion of the users and the mean costs calculated for the entire sample.

Overall, our results are surprisingly similar to the results in the European study, indicating that the disease impact is the same regardless of the continent. Differences stem essentially only from differences in the offer of care, in particular services such as paramedical professionals and services, and different prices of services. Both are largely consequences of differences in the wealth of countries. Total costs also depend crucially on the level of severity in sample. It is thus not possible to compare costs in Brazil to other studies. In particular, it is noticeable that—although costs increase with disease severity as in the European countries, the increase is much less steep: while costs in Europe triple between EDSS 0 and EDSS 6 and increase more than fivefold at EDSS 9, costs in the Brazilian study are only increase two-fold at EDSS 6 and thereafter are flat. This appears to be mostly due to much fewer services offered to disabled people in Brazil than in most European countries. However, the number of participants with severe disease was limited and our results have to be considered with caution. On the other hand, the cost distribution with the EDSS categories is very similar to Europe, with DMDs dominating costs in early and production losses and informal care in late disease.

The largest individual cost is as expected represented by DMDs, used by 58% of the sample and representing 40.3% of total societal costs. This is similar to Eastern Europe (Czech Republic, Hungary, Poland) where the cost of DMDs range from 63% to 79% of total costs in mild disease and from 27%-39% in moderate disease.

On the other hand, production losses appear low compared to other studies where indirect costs represented a majority of costs. Partly this is due to the population in our study with early disease and thus a larger proportion still in the work force; partly it may also be due to the particular method used for assessing production losses.

Fatigue and cognitive difficulties are similar: Fatigue was reported by 95% of patients both in Europe and in Brazil, and cognitive difficulties by 71% in Europe and 69% in Brazil. Utility decreased in a similar fashion in both studies, with a flattening of the curve in the middle range of disability due to the non-linearity of the EDSS scale. However, scores in mild disease are lower in Brazil, starting at 0.77 compared to around 0.9 in Europe [12]. This may be the effect of methodological differences in the Brazilian tariff compared to the original tariff used in the European study, but could also be due to a difference in how MS is regarded in the country. A study published in 2015 by Takemoto et al estimated utility in a multicenter, cross-sectional survey conducted in eight clinical centers in the South and Southeast regions of Brazil. The mean utility score in the sample was 0.59, and mean scores by disease severity were 0.74, 0.53 and 0.39, for the groups at EDSS 0–3, EDSS 4–6.5 and EDSS 7–9, respectively [10]. This is very similar to our results, with a mean score of 0.58 for the sample and 0.68, 0.48, 0.25 for the three severity groups.

## Conclusions

This is the first such large burden of illness survey in Brazil. The study confirms overall findings in other similar surveys, in particular as far as the burden of the disease on patients is concerned. Costs are as expected lower than in the 16 European countries in this international study, but the overall structure of the costs (increasing with increasing disability, dominated by DMD treatment in early disease and by production losses and informal care in late disease) is similar.
